# Privacy and Usability in COVID Enrollment Apps

**DOI:** 10.1093/geroni/igab046.2488

**Published:** 2021-12-17

**Authors:** Laura Calloway, Lesa Huber, L Jean Camp

**Affiliations:** 1 Indiana University, Bloomington, Indiana, United States; 2 School of Public Health, Indiana Univ, Bloomington, Indiana, United States

## Abstract

Enrollment apps for COVID-19 vaccinations are meant to be privacy-enhancing, but poor design puts privacy at risk. We report on a qualitative exploration of the experiences of older adults attempting to register for vaccination. We engaged in a think-aloud protocol with six participants over age 65 over Zoom as they used the New York state vaccination portal. Authentication requirements were: Medicare ID, DOB, address, and phone (optional). For this cohort, Social Security numbers were the default Medicare ID. We found that a privacy-enhanced authentication option exists, but efforts to use privacy-preserving enrollment were confounded by security-enhancing timeouts. Choosing to use the time-consuming privacy-preserving authentication increased the risk that available vaccines were taken. As a result, older adults reliant on volunteers to enroll revealed sensitive information and risked identity theft. A design that was meant to be privacy-enhancing by offering multiple avenues for authentication and ensuring logout via timeouts created a system where the more secure option was not effectively available due to a competing security mechanism. This was exacerbated by a counter counting down the number of vaccine sites available, similar to a well-known stress condition used to create cognitive load in laboratory experiments. All six participants used privacy-sensitive information to enroll; provided adequate information for identity theft; and all six encountered stop points. The countdown of available vaccination sites, the time required for insurance validation as an alternative to Medicare ID, and logging off after inactivity to prevent session theft each are good practices; but fail together.

